# Compound traditional Chinese medicine dermatitis ointment ameliorates inflammatory responses and dysregulation of itch-related molecules in atopic dermatitis

**DOI:** 10.1186/s13020-021-00555-7

**Published:** 2022-01-04

**Authors:** Rongrong Zhang, Hongyin Zhang, Shuai Shao, Yingxin Shen, Fengqin Xiao, Jiaming Sun, Songlan Piao, Daqing Zhao, Guangzhe Li, Mingming Yan

**Affiliations:** 1 Changchun University of Chinese Medicine, Changchun, Jilin China; 2 Jilin Provincial Science and Technology Innovation Center of Health Food of Chinese Medicine, Jilin Ginseng Academy, Changchun University of Chinese Medicine, Changchun, Jilin China

**Keywords:** CTCMDO, Medicinal substance, Atopic dermatitis, MAPK and NF-κB pathways

## Abstract

**Background:**

Atopic dermatitis (AD) is a chronic inflammatory skin disease accompanied with itchy and scaly rash. Compound traditional Chinese medicine dermatitis ointment (CTCMDO) consists of a mixture of extracts from five plants, which had been used in AD treatment due to good anti-inflammatory and anti-allergic effects.

**Materials and methods:**

In this study, high-performance liquid chromatography (HPLC) and liquid chromatography/mass spectrometer (LC/MS) were performed to analyze the active ingredients of CTCMDO in detail and to establish its HPLC fingerprint. Furthermore, the anti-inflammatory and antipruritic activities of CTCMDO were studied in the treatment of DNCB-induced AD in mice.

**Results:**

A total of 44 compounds including phenylpropionic acid compounds, alkaloid compounds, curcumin compounds and lignans were identified via combined HPLC and LC/MS. A fingerprint with 17 common peaks was established. In AD-like mice, DNCB-induced scratching behavior had been suppressed in the treatment of CTCMDO in a dose-dependent manner. Furthermore, the detailed experimental results indicated that the AD can be effectively improved via inhibiting the production of Th1/2 cytokines in serum, reversing the upregulation of substance P levels of itch-related genes in the skin, and suppressing the phosphorylation of JNK, ERK, and p38 in the skin.

**Conclusion:**

This work indicated that CTCMDO can significantly improve AD via attenuating the pathological alterations of Th1/2 cytokines and itch-related mediators, as well as inhibiting the phosphorylation of mitogen-activated protein kinase (MAPK) and nuclear factor-kappa B (NF-κB).

## Introduction

Atopic dermatitis (AD) is a common chronic inflammatory skin disease that often causes other allergic disorders, such as food allergies, asthma, and rhinitis [1]. Although AD is not life-threatening, frequent complications such as itchiness, sleep deprivation, social embarrassment and depression often occur due to visible skin lesions, which may seriously affect the physical and mental health of patients [2, 3]. It is well known that the imbalance of innate and adaptive immunity is the main cause of AD. AD mainly involves a dysregulation of the T-cell-mediated adaptive immunity system [4, 5]. Abnormal secretion of T-helper (Th) 1 and Th2 cytokines is one of the main causes of AD. In particular, interleukin (IL)-4 and IL-6 are the triggers of AD [6], which can promote the proliferation of B and T cells, synthesis of immunoglobulin E (IgE), and induction of Th2 differentiation. The previous studies had shown that the MAPK and NF‑κB pathways are important indicators of inflammatory regulation [7]. Furthermore, overexpression of phosphorylation of ERK, JNK, p38, and p65 may accelerate the AD symptoms [8]. The current strategies in the treatment of AD include topical application of emollients, corticosteroids, calcineurin inhibitors, phototherapy, oral administration of steroids and immunosuppressants, and biologics [9]. However, these treatment modalities usually accompanied with side effects and drug tolerance. Traditional Chinese medicine (TCM) as an alternative medicine has been widely used in treating various skin diseases in a long history due to its good advantages in a rapid recovery and less trauma [10–12].

Substance P (abbreviated as SP) as a highly active neuropeptide is widely distributed in the nervous system and other peripheral tissues and organs, and exhibits a range of physiological functions [13]. In the skin, SP is mainly released from sensory nerve endings, which participates in the local immune and inflammatory response of the skin through chemotaxis of immune cells, regulation of cytokine production, and relaxation of micro-vessels. Furthermore, SP can also promote the division and proliferation of a variety of cells in the skin, the synthesis of cell DNA, and repair of skin trauma [14]. As an important itching intermediary, SP is related to the occurrence of a range of itching skin diseases [15]. The effects of SP on the skin immune and inflammatory response are mainly reflected in three aspects, namely, chemotaxis of immune cells, regulation of the production of cytokines, and relaxation of micro-vessels. SP attracts T cells, monocytes/macrophages, and eosinophils. SP is also a chemokine of neutrophils, at a concentration of 10^–9^ mol/L, SP can directly affect their migration to inflammatory sites [16]. In addition, it is well known that SP released by peripheral sensory nerves plays an important role in the process of wound healing, which can participate in local inflammatory reactions and immune responses, to further promote wound healing [17].

Compound traditional Chinese medicine dermatitis ointment (CTCMDO), consists of oil extracts from six plants (*Coptis chinensis* Franch., *Phellodendron chinense* Schneid., *Angelica sinensis* (Oliv.) Diels, *Rehmannia glutinosa* Libosch., *Curcuma longa* L., and Sesame oil), has been used in treating AD as a novel medication. Specially, six individual ingredients in CTCMDO have shown anti-inflammatory and anti-allergic activity. In this prescription, *C. chinensis* Franch. contains a large number of alkaloids such as berberine and coptisine, which shows broad-spectrum antiviral, antibacterial effects, anti-inflammatory and analgesic effects [18]. *P. amurense* Rupr. mainly contains berberine and phellodendrine, which exhibits anti-inflammatory and antifungal effects [19]. Curcumin is the main component of *C. longa*, which can improve microcirculation and promote blood circulation [20]. The active ingredient of *A. sinensis* is ferulic acid, which can improve local microcirculation and inhibit platelet adhesion, and exhibits analgesic, anti-inflammatory, and anti-bacterial activities in vitro [21]. CTCMDO is the most prescribed drug in dermatology at the Affiliated Hospital of Changchun University of Chinese Medicine, it has been clinically used for more than 85% of outpatients in the treatment of AD, rash, and other inflammatory skin diseases.

Although the efficacy of CTCMDO has been widely recognized by doctors and patients, the underlying treating mechanism on AD is still unclear. In this study, to clarify the pharmacodynamics material basis of CTCMDO and to understand the underlying mechanisms on AD, HPLC, HPLC–MS, fingerprinting were performed to systematically analyze the compound composition of CTCMDO. In vivo, a DNCB-induced AD-like mouse model was established to investigate the therapeutic effect of CTCMDO against AD. Furthermore, the action pathway of CTCMDO in the treatment of AD was also investigated via molecular docking. All the results indicated that CTCMDO can significantly improve AD via attenuating the pathological alterations of Th1/2 cytokines and itch-related mediators, as well as inhibiting the phosphorylation of MAPKs.

## Materials and methods

### Herb materials

Coptidis Rhizoma (*C. chinensis* Franch.), Phellodendri Chinensis Cortex (*P. chinense* Schneid.), Angelicae Sinensis Radix (*A. sinensis* (Oliv.) Diels), Rehmanniae Radix (*R. glutinosa* Libosch.), Curcumae Longae Rhizoma (*C. longa* L.), and sesame oil (*Sesamum indicum* L.) were purchased from Jilin Xiancao Medicine Co., Ltd. Identification and authentication of the herb materials were carried out at Changchun University of Chinese Medicine, and voucher specimens were deposited at the Jilin Ginseng Academy, Changchun University of Chinese Medicine, Jilin, China.

### Chemicals and reagents

Rabbit anti-ERK1 + ERK2 antibody (bs-0022R), rabbit anti-phospho-ERK1/2 antibody (bs-3016R), rabbit anti-p38 MAPK antibody (bs-0637R), rabbit anti-Phospho-p38 MAPK (Thr180 + Tyr182) antibody (bs-0636R), rabbit anti-JNK1 + JNK2 + JNK3 (bs-2592R) and rabbit anti-phospho-JNK1 + JNK2 + JNK3 (T183 + T183 + T221) (bs-4163R) antibody were obtained from Beijing Biosynthesis Biotechnology Co., Ltd. Compound dexamethasone acetate cream was obtained from Guangzhou Baiyunshan Pharmaceutical Group Co., Ltd. Curcumin, sesamin, berberine, coptisine hydrochloride, phellodendrine, and ferulic acid standards with a purity greater than 98% were obtained from the China National Institute for the Control of Pharmaceutical and Biological Products, Beijing, China. Assay kits for IgE, IL-4, tumor necrosis factor (TNF)-α, IL-6, IL-2, and interferon (INF)-γ were purchased from RD, USA.

### Animals

BALB/c female mice (18–22 g) were purchased from the Hongda Biotechnology Co., Ltd, Changchun, Jilin, China (Certificate: No. SCXK (Liao) 2015–0001) and acclimatized for 7 days. They were kept under controlled ambient temperature (24 ± 2 °C) and humidity (60 ± 10%) with a 12-h light–dark cycle. Water was available ad libitum. The animal protocol was approved by the Animal Care Ethics Committee of Changchun University of Chinese Medicine (Changchun, Jilin, China, Approval No. 20181011).

### Preparation of CTCMDO and detection solution

The prescription of CTCMDO was composed of five herbs including Coptidis Rhizoma (4.5 g), Phellodendri Chinensis Cortex (4.5 g), Angelicae Sinensis Radix (7.5 g), Rehmanniae Radix (15 g), and Curcumae Longae Rhizoma (4.5 g). The above five herbs were ground to pieces and mixed together. The mixture of these herbal medicines was extracted with sesame oil (180 g) for 45 min at 120 °C, and removed the herb residue via hot filtration. The hot oil extract mixed with beeswax (*Apis cerana* Fabricius) according to the weight ratio of 3:1, stirred to completely dissolved, and then cooled down at room temperature to obtain the CTCMDO.

12 g of CTCMDO were accurately weighed and placed into a Soxhlet extractor. Petroleum ether was added and refluxed until the solution was colorless. Then replaced petroleum ether with equal amount of methanol, refluxed until methanol was colorless, then collected methanol extract. The methanol extract was dried under Rotavapor R-100 (BUCHI, Switzerland), then dissolved extract in 2 mL methanol (HPLC grade) and filtered through a 0.22 mm-membrane before detection.

### UHPLC‑MS/MS for the identification of ingredients in CTCMDO

Chromatographic analysis was performed using an UltiMate 3000 RS (Thermo Scientific, Sunnyvale, CA, USA) system equipped with a quaternary pump, vacuum degasser, auto sampler, and diode array detector. The mobile phase was 1% chromatographic formic acid aqueous solution (A) and 0.1% formic acid acetonitrile (B). The detailed mobile phase procedure is shown in Table 1. The chromatographic column was a C18 column (150 × 2.1 mm 1.8 µm) with a temperature of 35 °C, the injection volume was 10 μL, and the flow rate was 0.3 mL/min.

**Table 1:** Gradient elution program table

Time (min)	0.1% Aqueous formic acid (%) (A)	0.1% Formic acid acetonitrile (%) (B)
0	98	2
1	98	2
5	80	20
10	50	50
15	20	80
20	5	95
25	5	95
26	98	2
30	98	2

The Q-Exactive high-resolution mass spectrometer (Thermo Scientific, Sunnyvale, CA, USA) equipped with an electrospray ionization source was used in the positive ion mode and the negative ion mode, controlled by the Thermo Fisher software system. The automatic MS2 mode of the mass spectrometer was selected to analyze the sample. The following operating parameters were used: spray voltage: 3.8 kV (positive), capillary temperature: 300 °C, collision gas: high-purity argon (purity ≥ 99.999%), sheath gas: nitrogen (purity ≥ 99.999%), 40 Arb, auxiliary gas (Aux gus heater temp): nitrogen (purity ≥ 99.999%), 350 °C, data collection time: 30.0 min, scan range: 150.0–2000.0 m/z. The recorded data were processed using the applied Thermo Xcalibur software system.

### Establishment of the CTCMDO fingerprint

High-performance liquid chromatography (HPLC) analysis of the CTCMDO sample solution was performed on an Agilent 1260 Infinity HPLC system (Agilent, Santa Clara, CA, USA) equipped with a UV detector. Chromatographic separation was conducted on a Sepax Bio-C18 column (4.6 × 250 mm, 5 μm, from Sepax Technologies, Delaware, USA). The solvent system was composed of solvent A (acetonitrile) and solvent B (0.1% phosphoric acid in water) (Table 2). The operating conditions were as follows: detection wavelength, 278 nm, flow rate, 1.0 mL/min, column temperature, 30 °C, injection volume, 20 μL. Ten batches of CTCMDO were prepared and measured separately to identify the phytochemical constituents.

**Table 2:** Gradient elution schedule

Time (min)	0.1% Phosphate water (A) %	Acetonitrile (B) %
0 ~ 30	90 ~ 85	10 ~ 15
30 ~ 40	85 ~ 70	15 ~ 30
40 ~ 50	70 ~ 55	30 ~ 45
50 ~ 75	55	45

### Docking procedure

Molecular docking is a method of placing the ligand in the binding area of the receptor through computer simulation and calculating its physical and chemical parameters to predict the binding affinity and conformation of the ligand and receptor. The System Dock Web Site server (http://systemsdock.unit.oist.jp/iddp/home/index) evaluates the ligand–receptor binding potential through the molecular docking function of the docking score. Four targets with the highest degree value were selected, and they were imported into the System Dock Web Site server and then molecular docked with the active ingredients of CTCMDO. The results were obtained and evaluated by analysis of the docking score [22, 23].

### Preparation of CTCMDO in different administration groups

According to the previous description in ‘Preparation of CTCMDO and detection solution’, a half-dose prescription, a single-dose prescription and a double-dose prescription were taken into Sesame oil (180 g), respectively. Subsequently prepared ointment according to ‘Preparation of CTCMDO and detection solution’, CTCMDO-L (low-dose group, specifications of CTCMDO: 75 mg/g, crude drug/ointment), CTCMDO-M (medium-dose group, specifications of CTCMDO: 150 mg/g, crude drug/ointment), CTCMDO-H (high-dose group, specifications of CTCMDO: 300 mg/g, crude drug/ointment) were obtained, respectively. In the following animal experiments, the ointment was prepared on the basis of daily application dose for future use.

### Animal’s models and treatment

The establishment of the DNCB-induced AD model mice was based on previous publication with minor modification [24]. Mice (n = 10) were divided into the following 6 groups: NOR group (the normal group, free access to food and water without other treatment), DNCB (DNCB-sensitized group as a negative control group), DEX group (Compound Dexamethasone Acetate Cream group as a positive control group), CTCMDO-L, -M and -H group. Except the NOR group, the back skin area (2 cm × 2 cm) of the mouse was shaved with a tool, and the residual hair was removed by applying depilatory cream. from day -7 to day -4, 100 μL of 1% DNCB solution (dissolved in a 4:1 mixture of acetone and olive oil) for sensitization (for 3 days), then the mice were observed without any further treatment (for 4 days), from day 0 to day 18, all administration groups were treated with back application therapy, separately. The application dose of DEX and CTCMDO group in each administration group was 0.1 g/cm^2^ (ointment/back skin area, the ointment completely covered the wound).

### Determination of ear swelling

On the 17th day of administration treatment, the mice in each administration group were sensitized for the second time, 100 μL of 0.5% DNCB solution was applied to the ear skin. After 24 h, the mice were euthanized, the left and right ear biopsies were collected (about 8 mm) by a puncher and quickly weighed on an analytical balance, and the difference in weight between the ears was used as the swelling degree.

### ELISA for detection of the levels of cytokines (INF-γ, TNF-α, IL-6, IL-2, IL-4, IgE)

The total serum samples were collected 24 h after DNCB application on day 18, and the serum levels of INF-γ, TNF-α, IL-6, IL-4, IgE, and IL-2 were detected using mouse ELISA kits in accordance with the manufacturer’s instruction. Quantitation was done with an ELISA reader using 450 nm filters. Cytokine concentrations were calculated using a linear regression equation obtained from the standard absorbance values.

### Histological analysis

After the back skin was fixed in 10% formalin for 24 h, it was dehydrated and then embedded in paraffin and sliced (4-micron thickness). The sections of back skin were stained with hematoxylin and eosin (H&E) and observed under an optical microscope. The thickness of the epidermis and dermis and the number of inflammatory cells were measured using the Leica Application Suite. The magnification was × 200.

### Immunohistochemistry

The skin sections for immunohistochemistry were processed in the same manner as those for histological analysis. After deparaffinization and rehydration, the skin sections were boiled in 10 mM sodium citrate buffer for antigen retrieval. The slides were treated with 3% H_2_O_2_ for 30 min to reduce endogenous peroxidase and with normal goat serum (in PBS with 5% NHS, 5% fetal bovine serum, 2% bovine serum albumin (BSA), 0.1% Triton X-100) to minimize nonspecific binding. After blocking, the sections were incubated with antibodies against SP overnight at 4 °C. After washing with PBS, the sections were incubated with secondary anti-goat antibody for 1 h at room temperature. The sections were then stained using the Avidin/Biotinylated Enzyme Complex (ABC) Kit, and the substrate chromogen mixture was prepared immediately before use. Immunoreactivity was viewed with an LAS. The magnification was × 200.

### qRT-PCR analysis

After administration of CTCMDO for 17 days, the expression of p38 MAPK mRNA in mouse skin tissue was detected, and the total RNA of the mouse skin tissue was extracted by the TRIzol method for RT-PCR amplification. The p38 MAPK upstream primer was 5′-GATTGAGAT-GATTTTGGAG-3′ and downstream primer 5′-GTATGTTAAG-TATATGATTG-3′, and the amplified fragment was 482 bp. The PCR reaction conditions were as follows: 45 s at 94 °C, 50 s at 55 °C, 75 s at 72 °C, after 40 cycles to obtain the p38 MAPK mRNA cycle threshold (Ct), used ^ΔΔ^Ct (^ΔΔ^Ct = Ct target gene − ^Δ^Ct internal reference gene) analysis and calculated the relative expression of the 2^−ΔΔ^Ct target gene.

### Western blot analysis

The skin tissues of mice in each group were accurately weighed, and cut into small pieces with scissors, and extracted the protein from the tissues in accordance with the kit instructions to obtain the total protein of each group. A BCA kit was used to determine the protein concentration. Following 10% SDS-PAGE gel electrophoresis, the PVDF membrane was placed in 5% skimmed milk powder blocking solution for 1 h and washed three times with TBST, 5 min each time. Next, The primary antibodies (p65, p-p65, ERK, p-ERK, JNK, p-JNK, p38, and p-p38 primary antibodies diluted 1:1000) were diluted and incubated them at room temperature for 1 h. Then, washed the membrane by TBST for three times, 5 min each time, next, added diluted horseradish peroxidase-labeled secondary antibody for 1 h, washed the membrane three times, 5 min each time, followed by analysis using the Chemi Doc XRS system (Bio-Rad, Richmond, CA, USA).

### Statistical analysis

All of the data are expressed as the mean and standard deviation and represent three independent experiments. Statistical analyses were performed using SPSS 13.0. Treatment effects were analyzed using one-way analysis of variance (ANOVA) followed by Dunnett’s test. A value of P < 0.05 was used to indicate statistically significant differences.

## Results

### HPLC–MS analysis of CTCMDO

**Fig. 1:**
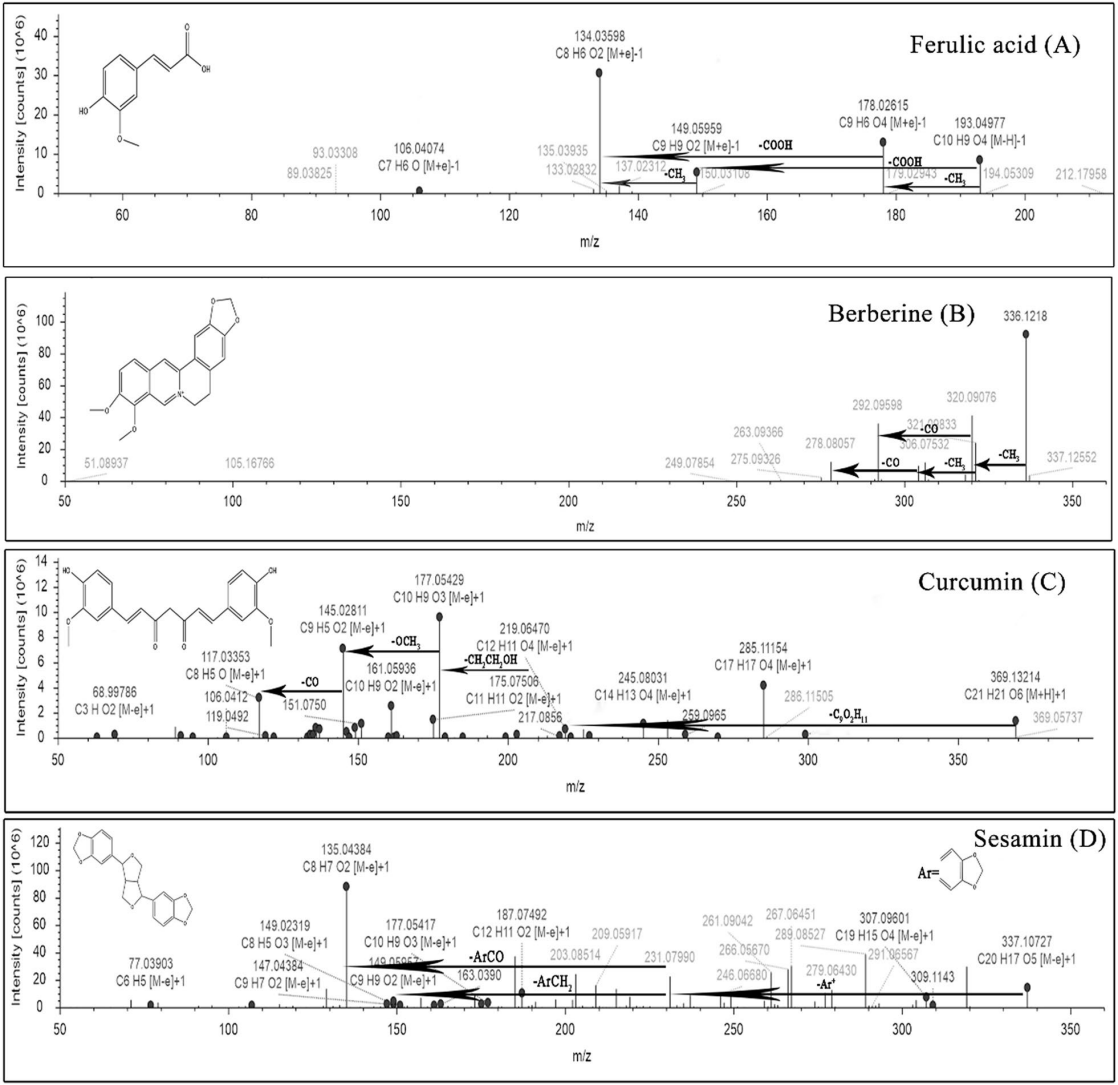
Targeted-MS/MS spectra of four identified compounds: ferulic acid (**A**), berberine (**B**), curcumin (**C**), and sesamin (**D**)

**Fig. 2:**
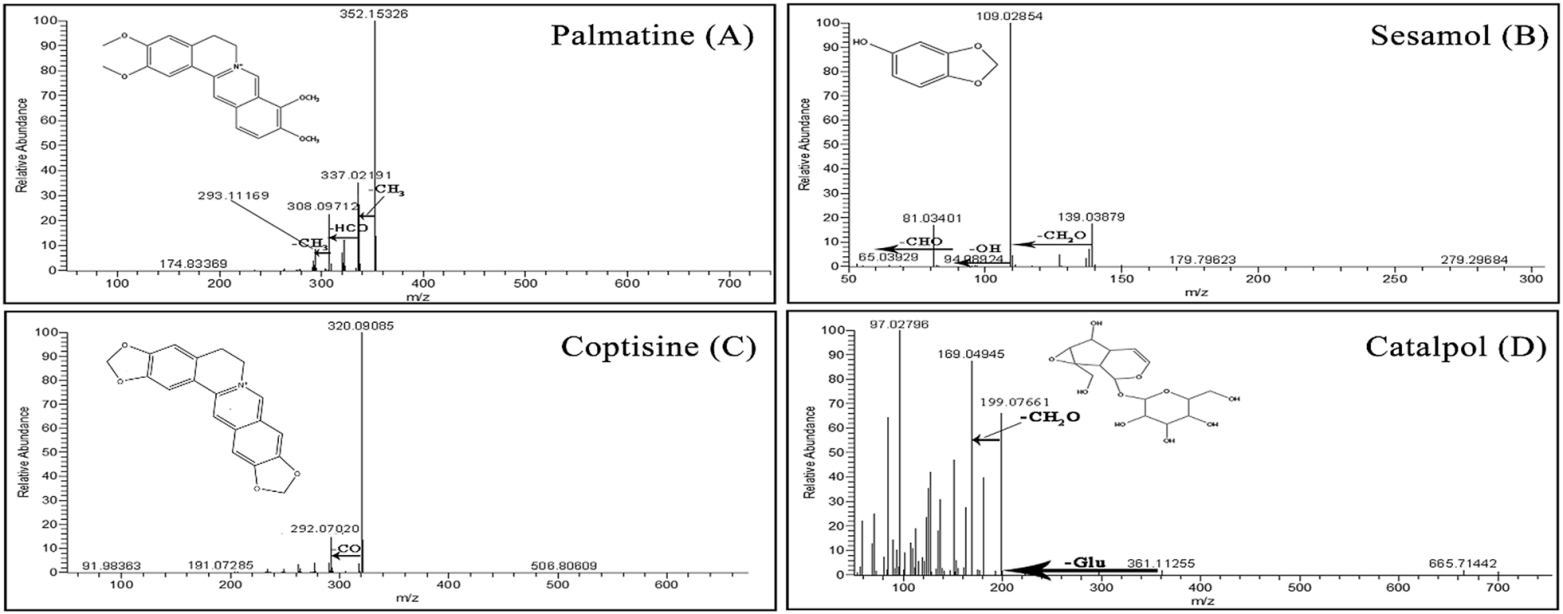
Targeted-MS/MS spectra of four identified compounds: palmatine (**A**), sesamol (**B**), coptisine (**C**), and catalpol (**D**)

HPLC–MS was firstly carried out to investigate the effective ingredients of CTCMDO, as shown in Table 3, totally 44 ingredients were identified. Finally, as shown in Figs. 1 and 2, berberine, palmatine, coptisine, phellodendrine, ferulic acid, sesamol, sesamin, catalpol and curcumin were confirmed via comparing the retention time of the commercially available reference substance, ultraviolet absorption spectrum and the high-resolution mass spectrum.

**Table 3:** Ultra-performance liquid chromatography-electrospray ionization time-of-flight mass spectrometry to identify the effective ingredients of CTCMDO

No.	RT	Name	Molecular formula	Theoretical molecular weight	Measured molecular weight	Fragment ion
1	0.5	Catalpol	C_15_H_22_O_10_	362.33	[M]-H] + 1 361.11255	361.1, 199.1, 169.05
2	1.16	Triethanolamine	C_6_H_15_NO_3_	149.10515	[M + H] + 1 150.11241	150.11, 132.1, 122.97, 58.07
3	1.453	Betaine	C_5_H_11_NO_2_	117.0792	[M + H] + 1 118.08654	118.09, 59.07, 58.07
4	1.488	D-Raffinose	C_18_H_32_O_16_	504.16871	[M + H] + 1 505.17612	527.16, 365.10, 203.05
5	1.772	Nicotinic acid	C_6_H_5_NO_2_	123.03228	[M + H] + 1 124.03950	124.04, 111.04, 79.05
6	3.49	phellodendrine	C_20_H_24_NO_4_	341.413	[M + H] + 1 342.16898	342.17, 192.1
7	4.26	Sesamol	C_7_H_6_O_3_	138.03115	[M + H] + 1 139.03879	109.03, 94.09, 65.04
8	4.27	Bis(2-ethylhexyl) phthalate	C_24_H_38_O_4_	390.2769	[M + H] + 1 391.28412	391.28, 226.89
9	4.50	Coptisine	C_19_H_14_NO_4_	337.2	[M−H_2_O]^+1^ 320.09085	318.09, 292.07
10	4.62	Palmatine	C_21_H_22_NO_4_	351.86	[M + H] + 1 352.15326	337.02, 322.02, 308.10, 293.11
11	5.839	5-Hydroxymethyl-2-furaldehyde	C_6_H_6_O_3_	126.03179	[M + H] + 1 127.03906	127.04, 109.03, 81.08, 57.03
12	9.47	3-Hydroxypicolinic acid	C_6_H_5_NO_3_	139.02699	[M + H] + 1 140.03427	140.03, 122.06, 95.09, 79.01
13	10.337	Puerarin	C_21_H_20_O_9_	416.1103	[M + H] + 1 417.11785	417.12, 399.11, 381.10
14	10.815	Berberine	C_20_H_17_NO_4_	335.11495	[M + H] + 1 336.12220	336.12, 306.08, 278.08
15	11.087	Daidzin	C_21_H_20_O_9_	416.11031	[M + H] + 1 417.11755	417.07, 237.05, 199.08, 181.06
16	11.372	Cyclo(phenylalanyl-prolyl)	C_14_H_16_N_2_O_2_	244.12105	[M + H] + 1 245.12827	245.13, 172.11
17	11.683	Ferulic acid	C_10_H_10_O_4_	194.05734	[M + H] + 1 193.04973	193.05, 178.03, 149.06, 134.04, 106.04
18	12.611	Senkyunolide H	C_12_H_16_O_4_	206.09422	[M + H] + 1 207.10150	207.10, 165.05, 123.04, 95.05
19	13.918	Daidzein	C_15_H_10_O_4_	254.05759	[M + H] + 1 253.05025	253.05, 235.13, 167.05
20	14.938	Curcumin	C_21_H_20_O_6_	368.12552	[M + H] + 1 369.13306	369.13, 299.13, 245.08, 177.05
21	15.041	Methyl palmitate	C_17_H_34_O_2_	287.28234	[M + H] + 1 287.28230	288.29, 270.28, 226.15
22	16.836	Sesamin	C_20_H_18_O_6_	336.09902	[M + H] + 1 337.10630	336.10, 307.10, 187.07, 135.04, 77.04
23	17.915	( +)-ar-Turmerone	C_15_H_20_O	216.15122	[M + H] + 1 217.15849	217.16, 125.10, 55.05
24	18.665	(-)-Caryophyllene oxide	C_15_H_24_O	220.18259	[M + H] + 1 221.18980	221.19, 203.18, 165.13
25	19.12	9-Oxo-10(E),12(E)-octadecadienoic acid	C_18_H_30_O_3_	294.21939	[M + H] + 1 295.22644	295.2, 241.19, 179.14, 151.11
26	19.27	α-Eleostearic acid	C_18_H_30_O_2_	278.22444	[M + H] + 1 279.23160	279.23, 261.22, 57.07
27	19.645	18-β-Glycyrrhetinic acid	C_30_H_46_O_4_	470.33923	[M + H] + 1 471.34674	471.35, 453.34, 425.34, 407.33
28	20.327	9(Z),11(E),13(E)-Octadecatrienoic Acid methyl ester	C_19_H_32_O_2_	292.23989	[M + H] + 1 293.24716	293.25, 243.21, 219.21, 55.06
29	20.418	Palmitoleic acid	C_16_H_30_O_2_	254.22429	[M + H] + 1 255.23157	255.23, 237.22, 195.17, 181.16
30	20.669	Palmitelaidic acid methyl ester	C_17_H_32_O_2_	268.23989	[M + H] + 1 269.24716	269.25, 135.12, 55.06
31	21.131	Palmitoyl ethanolamide	C_18_H_37_NO_2_	282.2556	[M + NH_4_] + 1 300.28934	300.29, 282.28, 239.24
32	21.137	1-Linoleoyl glycerol	C_21_H_38_O_4_	354.27647	[M + H] + 1 355.28375	355.28, 337.27, 133.10, 72.09
33	21.22	Hexadecanamide	C_16_H_33_NO	255.25567	[M + H] + 1 256.26294	256.26, 130.12, 102.09, 88.08, 57.07
34	21.424	16-Hydroxyhexadecanoic acid	C_16_H_32_O_3_	272.23518	[M + H] + 1 271.22791	271.23, 253.22, 225.22
35	21.482	Oleoyl ethanolamide	C_20_H_39_NO_2_	325.29778	[M + H] + 1 326.30505	326.31, 309.28, 247.24, 151.15
36	21.566	Oleamide	C_18_H_35_NO	281.27117	[M + H] + 1 282.27850	282.28, 212.20, 177.16, 114.09
37	21.871	Monoolein	C_21_H_40_O_4_	356.2922	[M + H] + 1 357.29947	357.30, 339.29, 247.24, 181.16
38	22.425	Stearamide	C_18_H_37_NO	283.2871	[M + H] + 1 284.29430	284.29, 144.14, 102.09, 57.07
39	22.872	Ricinoleic acid methyl ester	C_19_H_36_O_3_	294.25585	[M + H] + 1 295.26300	295.26, 245.23, 147.12, 55.06
40	23.48	Stearic acid	C_18_H_36_O_2_	284.27147	[M + H] + 1 283.26419	283.26, 270.63, 239.20
41	23.745	Erucamide	C_22_H_43_NO	337.33355	[M + H] + 1 338.34082	338.34, 303.30, 212.20, 149.13
42	25.012	Docosanamide	C_22_H_45_NO	339.34969	[M + H] + 1 340.35696	340.36, 69.07, 57.07
43	25.9	Nervonic acid methyl ester	C_25_H_48_O_2_	380.36467	[M + H] + 1 381.37195	381.37, 349.35, 331.33
44	17.45	Sesamolin	C_20_H_18_O_7_	352.0941	[M + H] + 1 353.08720	353.0872, 149.023, 77.04

### Fingerprint of CTCMDO

**Fig. 3:**
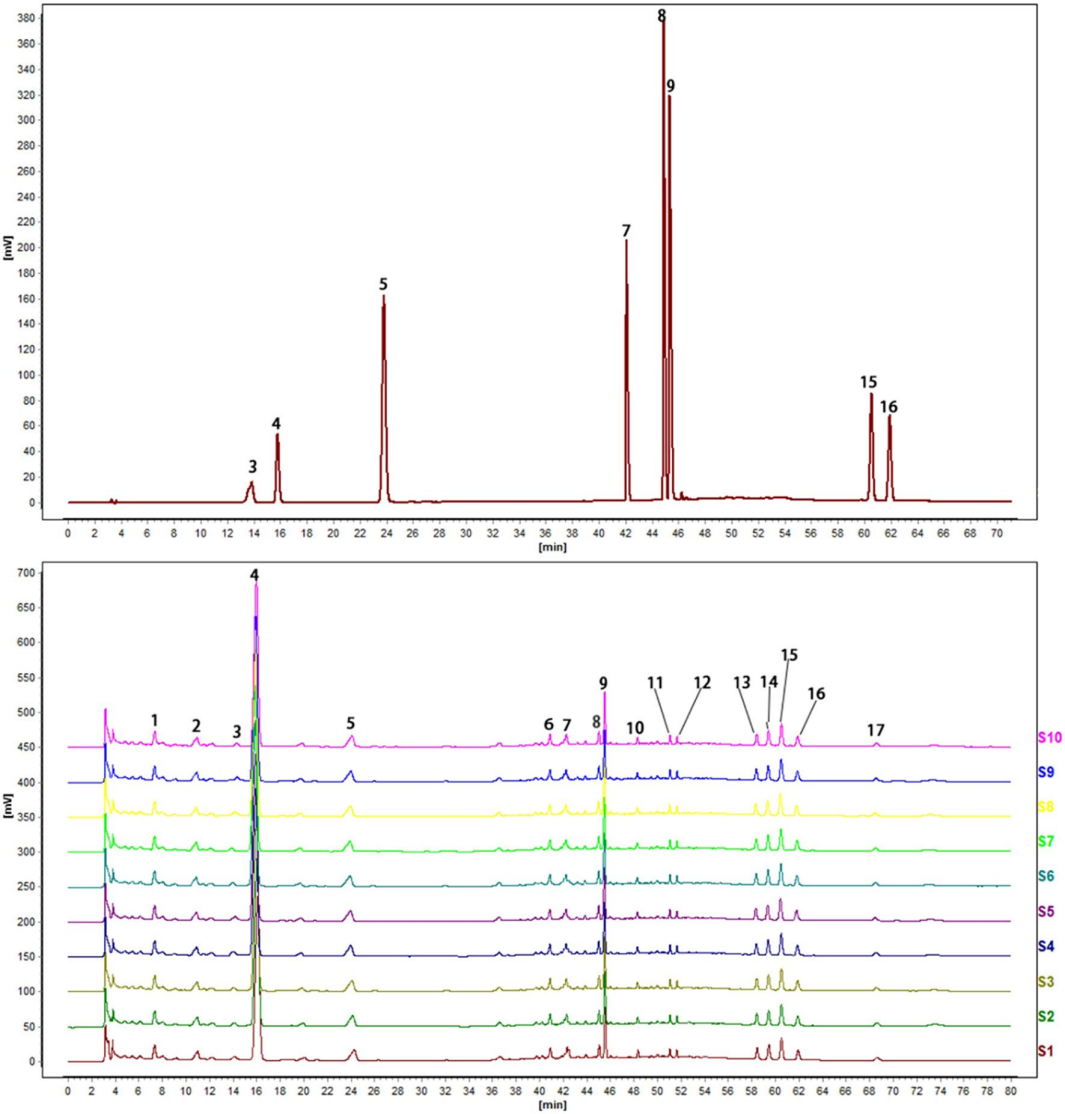
The fingerprint of CTCMDO. **A** Fingerprints of CTCMDO from 10 batches, **B** mixture of eight reference substances: 3. phellodendrine, 4. sesamol, 5. ferulic acid, 7. coptisine, 8. berberine, 9. palmatine, 15. curcumin, 16. sesamin

Ten batches of test solution were prepared to identify the active ingredients of CTCMDO. As shown in Fig. 3, totally 17 common peaks were determined. As shown in Table 4, eight ingredients including No. 3 phellodendrine, No. 4 sesamol, No. 5 ferulic acid, No. 7 coptisine, No. 8 berberine, No. 9 palmatine, No. 15 curcumin and No. 16 sesamin were identified via comparing the retention times of the chromatograms of the mixed standard reference. The above eight ingredients can be regarded as main active ingredients of CTCMDO due to the relevant studies had shown that the above eight components could exhibit anti-inflammatory effects in some extent [25, 26].

**Table 4:** Identification and determination of the compounds in CTCMDO by HPLC

Peak No.	Retention time (min)		Compounds	Contents (mg/mL)
	Reference standard	CTCMDO		
3	13.85 ± 1.65	14.15 ± 0.27	Phellodendrine	0.651 ± 0.0001
4	15.81 ± 0.65	16.08 ± 052	Sesamol	0.601 ± 0.0004
5	23.81 ± 1.63	24.26 ± 0.15	Ferulic acid	0.477 ± 0.002
7	42.09 ± 0.62	42.34 ± 0.03	Coptisine	0.349 ± 0.0009
8	44.89 ± 0.69	44.96 ± 0.12	Berberine	0.198 ± 0.001
9	45.34 ± 0.71	45.56 ± 0.32	Palmatine	0.319 ± 0.004
15	60.64 ± 0.61	60.51 ± 1.04	Curcumin	0.607 ± 0.005
16	61.92 ± 0.56	61.92 ± 0.16	Sesamin	0.871 ± 0.0003

The retention time and content of the compounds are expressed as the mean of three measurements (mean ± SD)

### Molecular docking

**Fig. 4:**
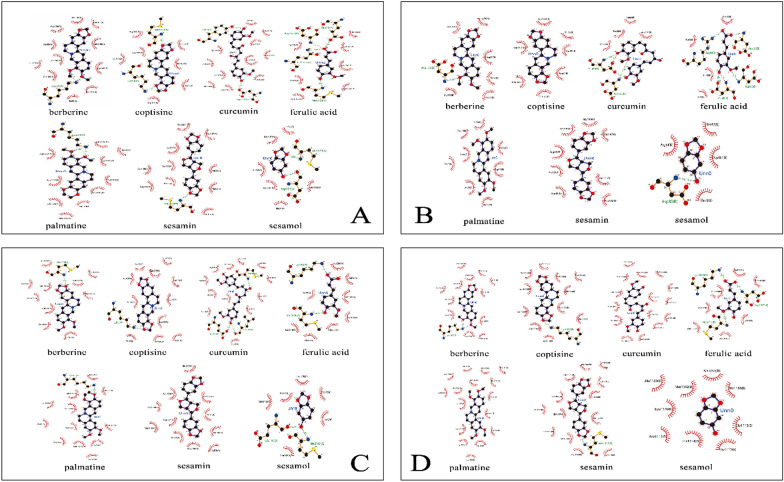
The two-dimensional docking analysis of four important targets of CTCMDO in the treatment of AD (ERK (**A**), JNK (**B**), NF-κB p65 (**C**), MAPK p38 (**D**)), dashed lines represent hydrogen bonds, and labeled residues represent hydrophobic interactions

The MAPK signaling pathway has been regarded as a key pathway for inflammatory diseases, which can transmit extracellular signals (such as stress and growth factors) into the cell, thereby affect TNF-α and IL-6 production. Other inflammatory factors for instance cyclooxygenase 2 (COX-2) had been proofed could participate in the regulation of cell proliferation, differentiation, and apoptosis [27]. TNF-α directly or indirectly participates in the NF-κB signaling pathway, the MAPK signaling pathway and many other signaling pathways, thereby formed complex interaction relationships [28]. Therefore, the MAPK signaling pathway and the NF-κB signaling pathway targets were selected for molecular docking verification. The target protein data file was downloaded through the PDB database, and GOLD software was selected to compare the fingerprints of the active ingredients of CTCMDO and the relevant target proteins. The molecular docking results are shown in Fig. 4.

The interactive activities of the CTCMDO compounds with the key enzymes of the MAPK pathway were examined via molecular docking analysis. The binding potency of these compounds with ERK (Fig. 4A), JNK (Fig. 4B), NF-κB p65 (Fig. 4C), and MAPK p38 (Fig. 4D) (Table 5) was tested, respectively. The calculation results indicated that seven components exhibited good binding ability among the above-mentioned eight components. Coptisine exhibited best combination ability with ERK (− 7.81 kcal/mol), and the order from strong to weak was as follows: sesamin > berberine > palmatine > curcumin > ferulic acid > sesamol. Sesamin exhibited best combination ability with JNK (− 8.24 kcal/mol), and the order from strong to weak was as follows: coptisine > palmatine > curcumin > berberine > ferulic acid > sesamol. Palmatine showed best combination ability with p65 (− 5.45 kcal / mol), and the order from strong to weak was as follows: coptisine > sesamin > ferulic acid > berberine > sesamol > curcumin. Coptisine exhibited best binding ability to MAPK p38 (− 9.05 kcal/mol), and the order from strong to weak was as follows: sesamin > berberine > palmatine > curcumin > ferulic acid > sesamol. All the results indicated that coptisine, sesamin, and palmatine of CTCMDO exhibited best binding ability to MAPK, which may play the main pharmacological role in regulating immune activity.

**Table 5:** Molecular docking scores of the main components of CTCMDO

Compounds	Score (Kcal/mol)			
	ERK	JNK	NF-κB p65	MAPK p38
Berberine	− 6.96	− 7.48	− 4.73	− 7.47
Coptisine	− 7.81	− 8.13	− 5.26	− 9.05
Curcumin	− 5.83	− 7.93	− 4.12	− 6.12
Ferulic acid	− 4.99	− 4.95	− 4.94	− 5.61
Palmatine	− 6.37	− 8.08	− 5.45	− 7.02
Sesamin	− 7.39	− 8.24	− 5.22	− 7.55
Sesamol	− 4.34	− 4.32	− 4.37	− 4.71

### Experimental validation

#### Effect of CTCMDO on an AD model in mice

**Fig. 5:**
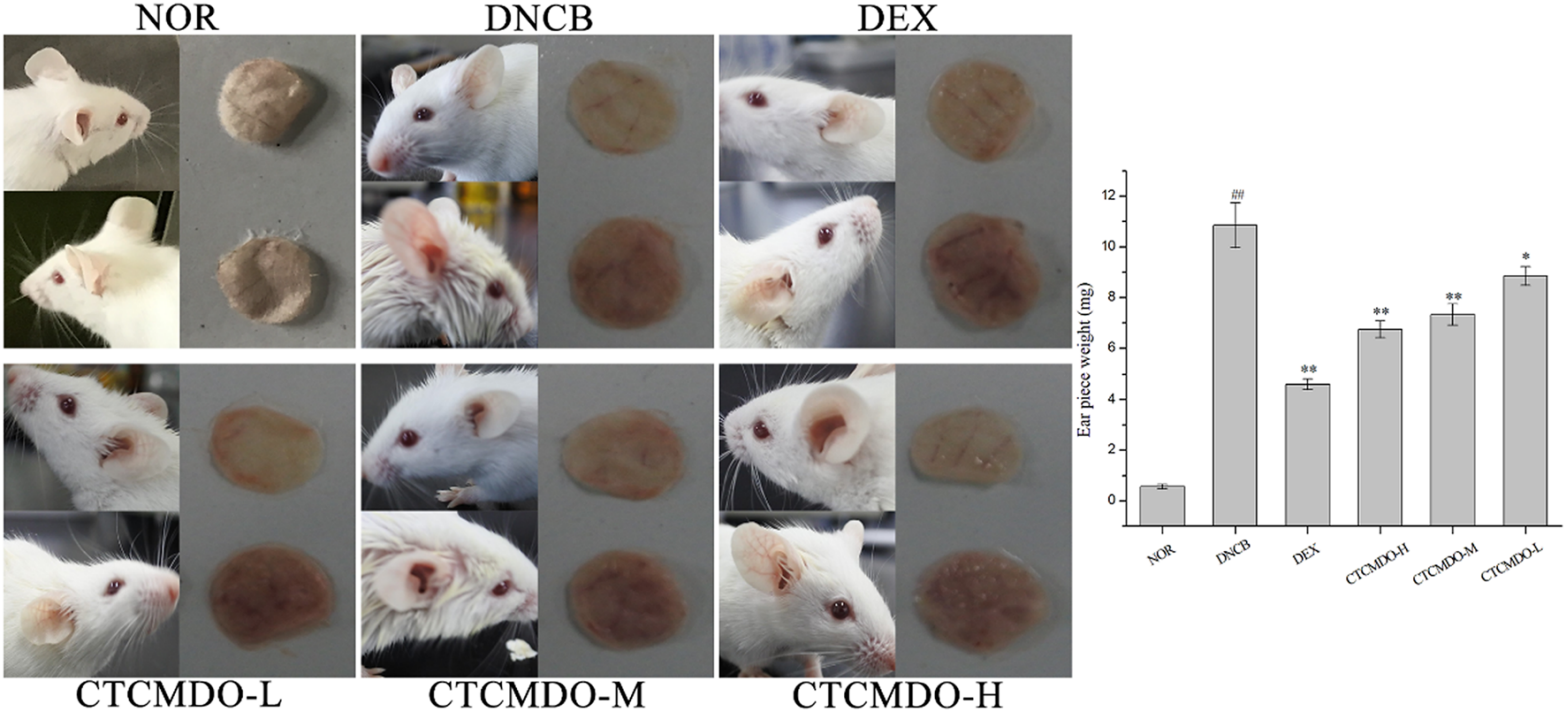
Effect of CTCMDO on ear swelling in mice. *NOR* normal control, *DNCB* DNCB-sensitized mice, *DEX* dexamethasone (0.1 g/cm^2^) + DNCB-sensitized mice. CTCMDO-L (75 mg/g, crude drug/ointment, 0.1 g/cm^2^) + DNCB-sensitized mice. CTCMDO-M (150 mg/g, crude drug/ointment, 0.1 g/cm^2^) + DNCB-sensitized mice and CTCMDO-H (300 mg/g, crude drug/ointment, 0.1 g/cm^2^) + DNCB-sensitized mice. Data are presented as mean ± SEM (n = 10). ##Significant difference from NOR group, P < 0.01. *p < 0.05 and **p < 0.01 versus DNCB group

The obviously different therapeutic effect in mice were observed when treated with CTCMDO at different concentrations. It is generally believed that the degree of ear swelling objectively reflects the degree of the inflammation response, and the ear swelling degree of each group of mice are shown in Fig. 5. Compared with the DNCB group, the DEX group showed a significant inhibitory effect on mouse ear swelling (p < 0.01), the CTCMDO-H and CTCMDO-M groups exhibited a significant inhibitory effect on mouse ear swelling (p < 0.01), and the ear swelling in mice was also improved in CTCMDO-L group (p < 0.05).

**Fig. 6:**
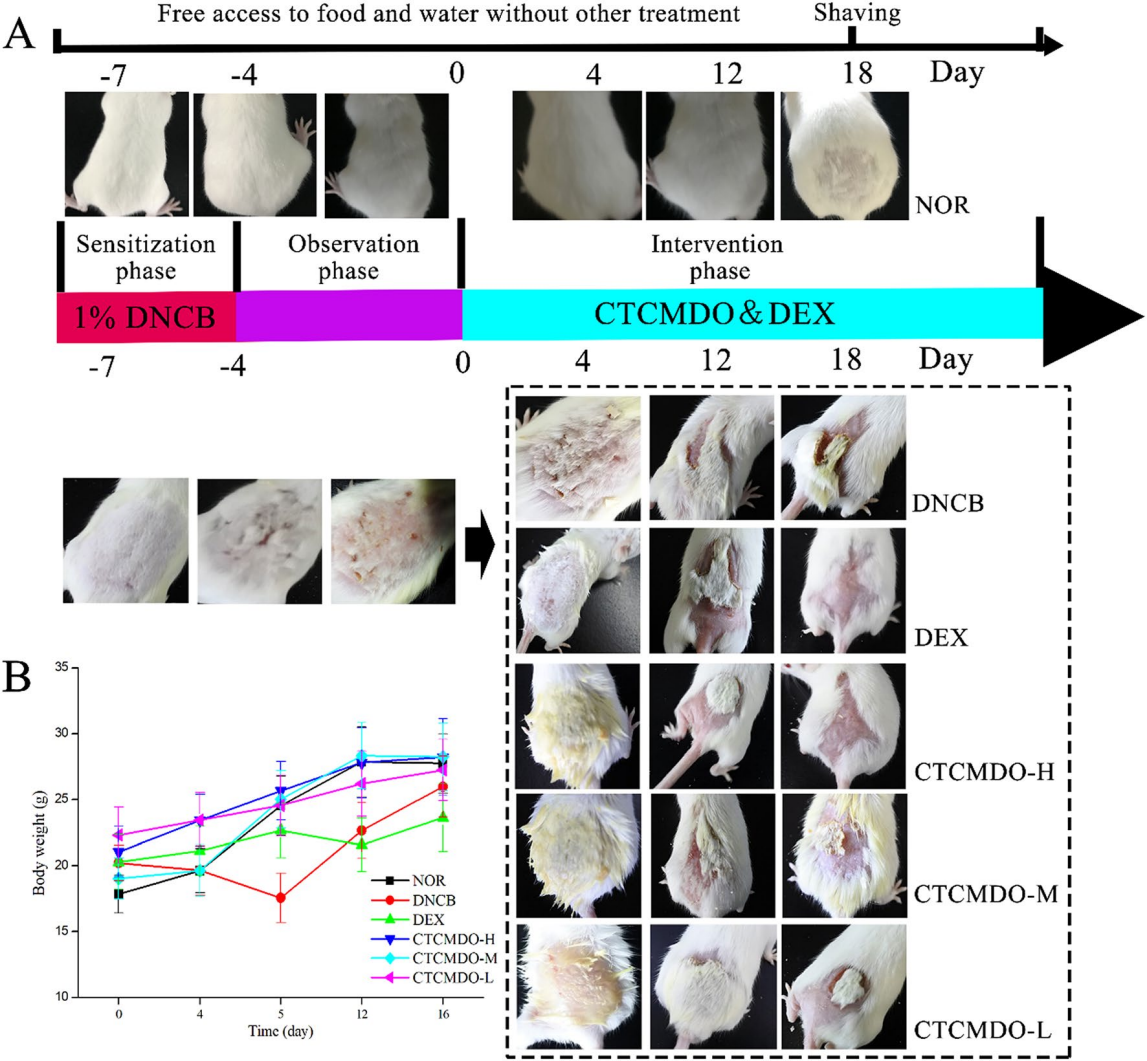
Wound-healing performance in different mice groups **A** the body weight in Administration groups was recorded every 4 days (**B**). *NOR* normal control, *DNCB* DNCB-sensitized mice, *DEX* dexamethasone (0.1 g/cm^2^) + DNCB-sensitized mice. CTCMDO-L (75 mg/g, crude drug/ointment, 0.1 g/cm^2^) + DNCB-sensitized mice. CTCMDO-M (150 mg/g, crude drug/ointment, 0.1 g/cm^2^) + DNCB-sensitized mice and CTCMDO-H (300 mg/g, crude drug/ointment, 0.1 g/cm^2^) + DNCB-sensitized mice. Data are presented as mean ± SEM (n = 10). ##Significant difference from NOR group, P < 0.01. *p < 0.05 and **p < 0.01 versus DNCB group

The skin healing abilities of each group of mice are shown in Fig. 6. After treated all mice with DNCB, the skin gradually thickened and crusted. After treatment with CTCMDO and DEX, the molting of the back of the mice gradually decreased until disappeared. In the DEX group and the CTCMDO-H group, molting was ceased completely at day 18. The body weight of individuals from all groups was recorded every 4 days. Combined with the behavioral observation and each group's weight data, it was found that the body weight of mice in the DEX group was significantly lower than that in the other dose groups (p < 0.01). Moreover, the mice in the DEX group showed delayed response when exposed to external stimuli.

**Fig. 7:**
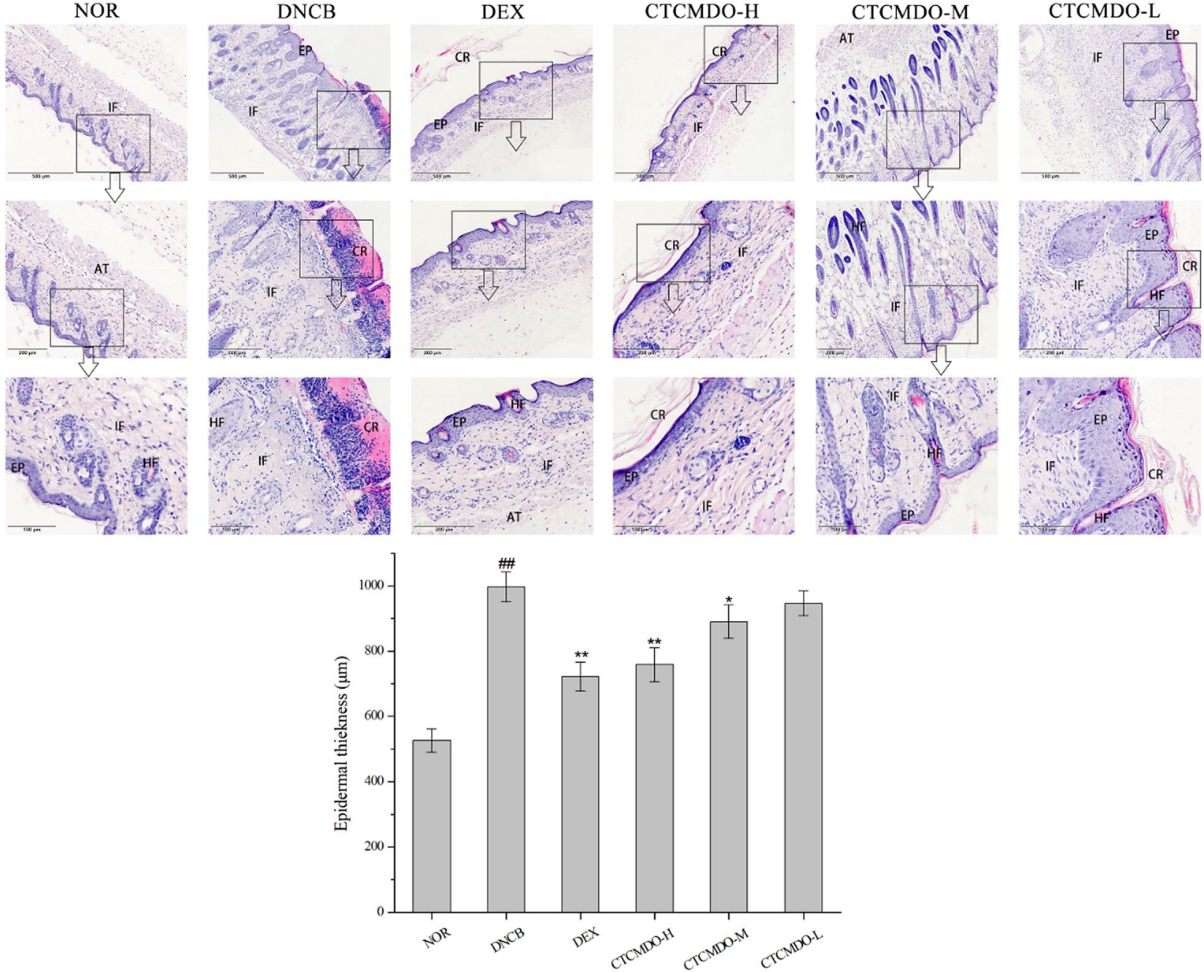
Effects of CTCMDO on the epidermal thickness in the dorsal skin. Dorsal skin sections were stained with H&E and analyzed for epidermal thickness (scale bar = 500 µm). *NOR* normal control, *DNCB* DNCB-sensitized mice, *DEX* dexamethasone (0.1 g/cm^2^) + DNCB-sensitized mice. CTCMDO-L (75 mg/g, crude drug/ointment, 0.1 g/cm^2^) + DNCB-sensitized mice. CTCMDO-M (150 mg/g, crude drug/ointment, 0.1 g/cm^2^) + DNCB-sensitized mice and CTCMDO-H (300 mg/g, crude drug/ointment, 0.1 g/cm^2^) + DNCB-sensitized mice. Data are presented as mean ± SEM (n = 10). ##Significant difference from NOR group, P < 0.01. *p < 0.05 and **p < 0.01 versus DNCB group

In order to evaluate the potential therapeutic ability of CTCMDO against AD, the histopathological changes of back skin of mice were investigated via H&E staining. Mice were treated with DEX and CTCMDO locally during the DNCB induction and sensitization phase. As shown in Fig. 7, compared with the normal group, the back skin of mice in the experimental model group (DNCB group) was significantly thickened and keratinized, and the thickness of the epidermis and the dermis significantly increased under the stimulation of DNCB (996.34 μm). The DEX and CTCMDO-H treatments significantly improved these pathological changes as reflected by the increasing in the thickness of the epidermis and dermis, compared with the DNCB group (DEX: 721.56 μm, CTCMDO-H: 758.49 μm). However, the effect of CTCMDO-L on skin thickness was not as expected, and only tiny change was observed in comparison with the DNCB group (P > 0.05). The above results showed that CTCMDO significantly inhibited the increase of epidermal and dermal thickness.

#### Inhibitory effects of CTCMDO on substance P expression

**Fig. 8:**
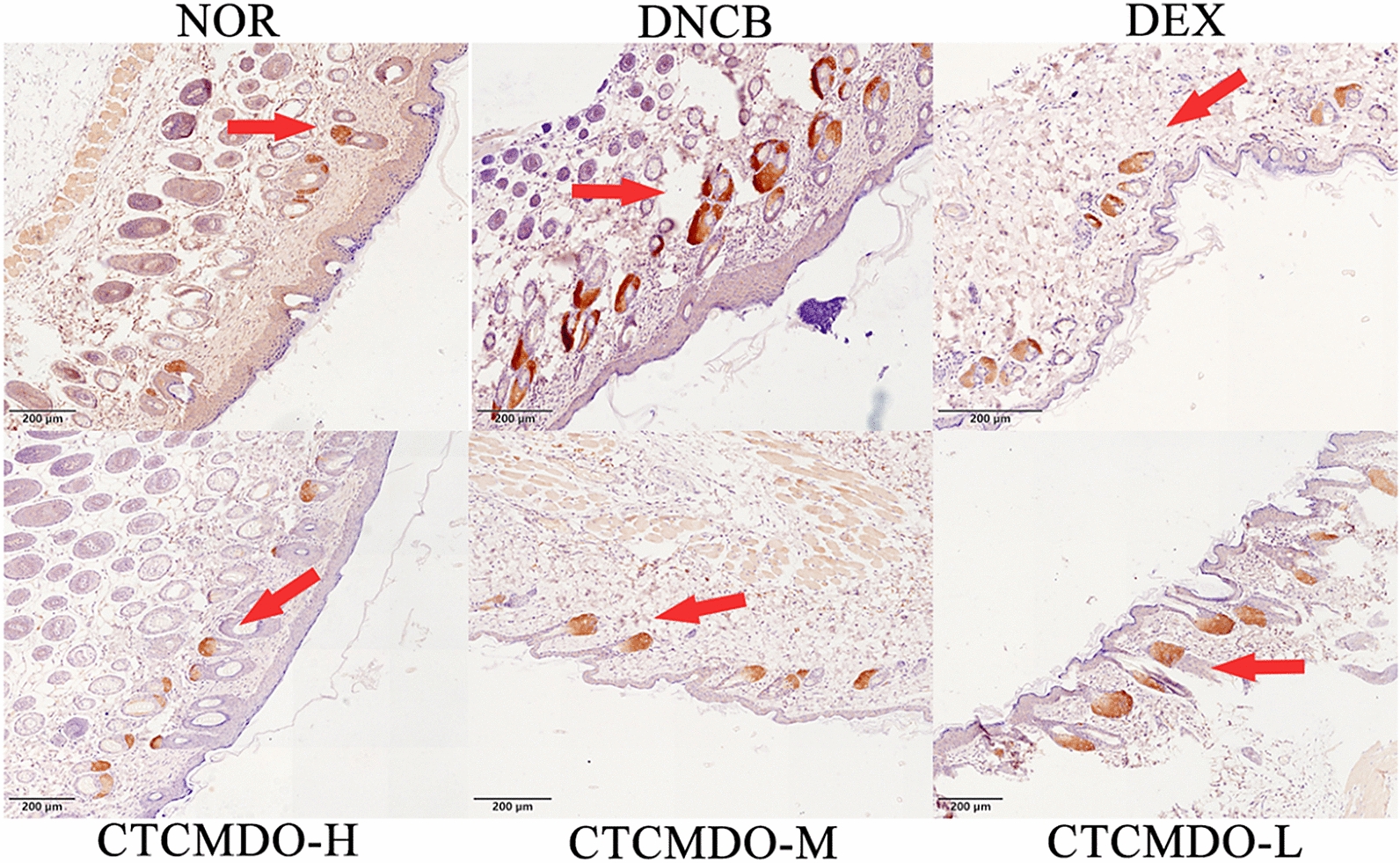
Effects of CTCMDO on DNCB-induced mice for substance P expression. (Marked with red arrow, scale bar = 500 µm)

Positive staining for SP is brown, which is mainly distributed in the cytoplasm or interstitium [29]. The immunohistochemistry results are shown in Fig. 8. DNCB group showed effective staining due to abundant SP. In contrast, repeated application of CTCMDO significantly inhibited the expression of SP. And the application of dexamethasone acetate also significantly inhibited the expression of SP. Compared with the NOR group, the expression of SP in the skin of the DNCB group showed more brown-yellow particles with darker coloration, and the positive expression was mainly located in the epidermis. The DEX group, CTCMDO-H group and -M group showed poor expression, and the NOR group showed lighter coloration in comparison with the DNCB group. Compared with the DNCB group, SP secretion was significantly inhibited in the DEX group, and the expression of SP were also significantly inhibited in both CTCMDO-H and -M groups. These results indicated that the expression of SP can be inhibited by CTCMDO, to further reduce the allergic reaction during the onset of AD [30].

#### Inhibitory effect of CTCMDO on cytokines

**Fig. 9:**
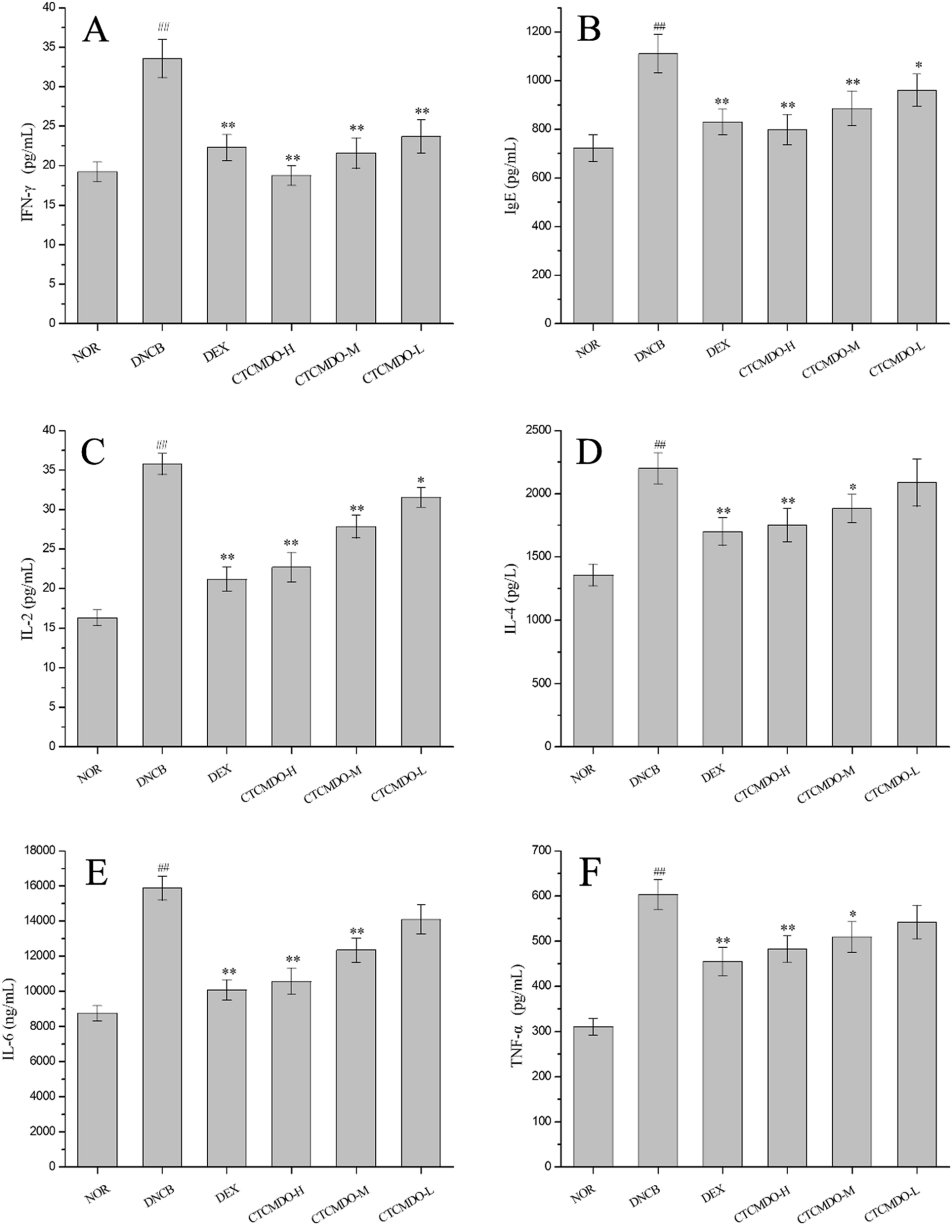
Effects of CTCMDO on DNCB-induced cytokine levels, including INF-γ (**A**), IgE (**B**), IL-2 (**C**), IL-4 (**D**), IL-6 (**E**), and TNF-α (**F**) in mice. *NOR* normal control. *DNCB* DNCB-sensitized mice. *DEX* dexamethasone (0.1 g/cm^2^) + DNCB-sensitized mice. CTCMDO-L (75 mg/g, crude drug/ointment, 0.1 g/cm^2^) + DNCB-sensitized mice. CTCMDO-M (150 mg/g, crude drug/ointment, 0.1 g/cm^2^) + DNCB-sensitized mice and CTCMDO-H (300 mg/g, crude drug/ointment, 0.1 g/cm^2^) + DNCB-sensitized mice. Data are presented as mean ± SEM (n = 10). ##Significant difference from NOR group, P < 0.01. *p < 0.05 and **p < 0.01 versus DNCB group

To evaluate the effect of CTCMDO on cytokine production, INF-γ, TNF-α, IL-6, IL-4, IgE and IL-2 were measured in serum. Compared with DNCB-induced mice, the levels of INF-γ, TNF-α, IL-6, IL-4, IgE and IL-2 were significantly inhibited when treated with CTCMDO or DEX (P < 0.05, P < 0.01, Fig. 9). Specifically, CTCMDO-H and -M groups showed significant changes in the secretion of related inflammatory factors and could significantly inhibit the secretion of all inflammatory factors (P < 0.01). CTCMDO-L group exhibited poor inhibitory effect on TNF-α, IL-6 and IL-4 at low dose, whereas it can be significantly improved when the dose was increased.

#### The expression of MAPK p38 mRNA in the skin tissue of each group of mice

**Fig. 10:**
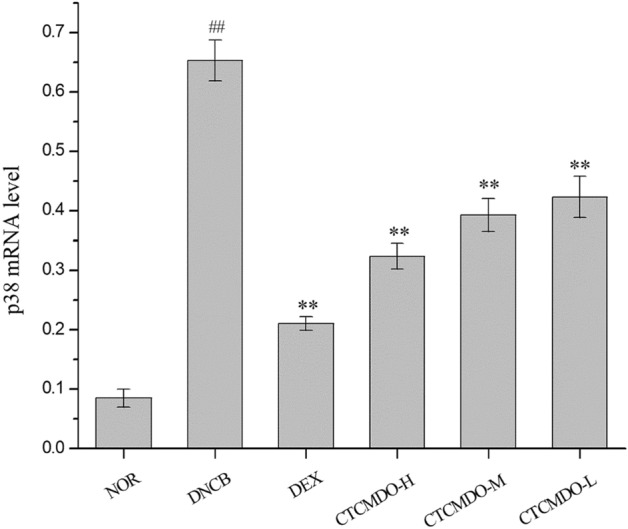
Effects of MAPK p38 expression in skin tissue of mice between the groups. *NOR* normal control, *DNCB* DNCB-sensitized mice, *DEX* dexamethasone (0.1 g/cm^2^) + DNCB-sensitized mice. CTCMDO-L (75 mg/g, crude drug/ointment, 0.1 g/cm^2^) + DNCB-sensitized mice. CTCMDO-M (150 mg/g, crude drug/ointment, 0.1 g/cm^2^) + DNCB-sensitized mice and CTCMDO-H (300 mg/g, crude drug/ointment, 0.1 g/cm^2^) + DNCB-sensitized mice. Data are presented as mean ± SEM (n = 10). **P < 0.01, *P < 0.05 compared with the DNCB group, ## P <  0.01 compared with the NOR group

In order to detect the changes of MAPK p38 mRNA in the dorsal skin of mice, qRT-PCR was used to detect the changes of p-p38 and p38 levels, and one-way ANOVA was used for comparative analysis. As shown in Fig. 10, all the results indicated that compared with the NOR group, the expression of p-p38 MAPK mRNA in the tissue was increased in the model group. Compared with the model group, the expression of p-p38 MAPK mRNA was significantly decreased in the skin tissue of the CTCMDO group, and the difference was statistically significant (P < 0.01).

#### Inhibitory effects of CTCMDO on MAPK/NF-κB expression

**Fig. 11:**
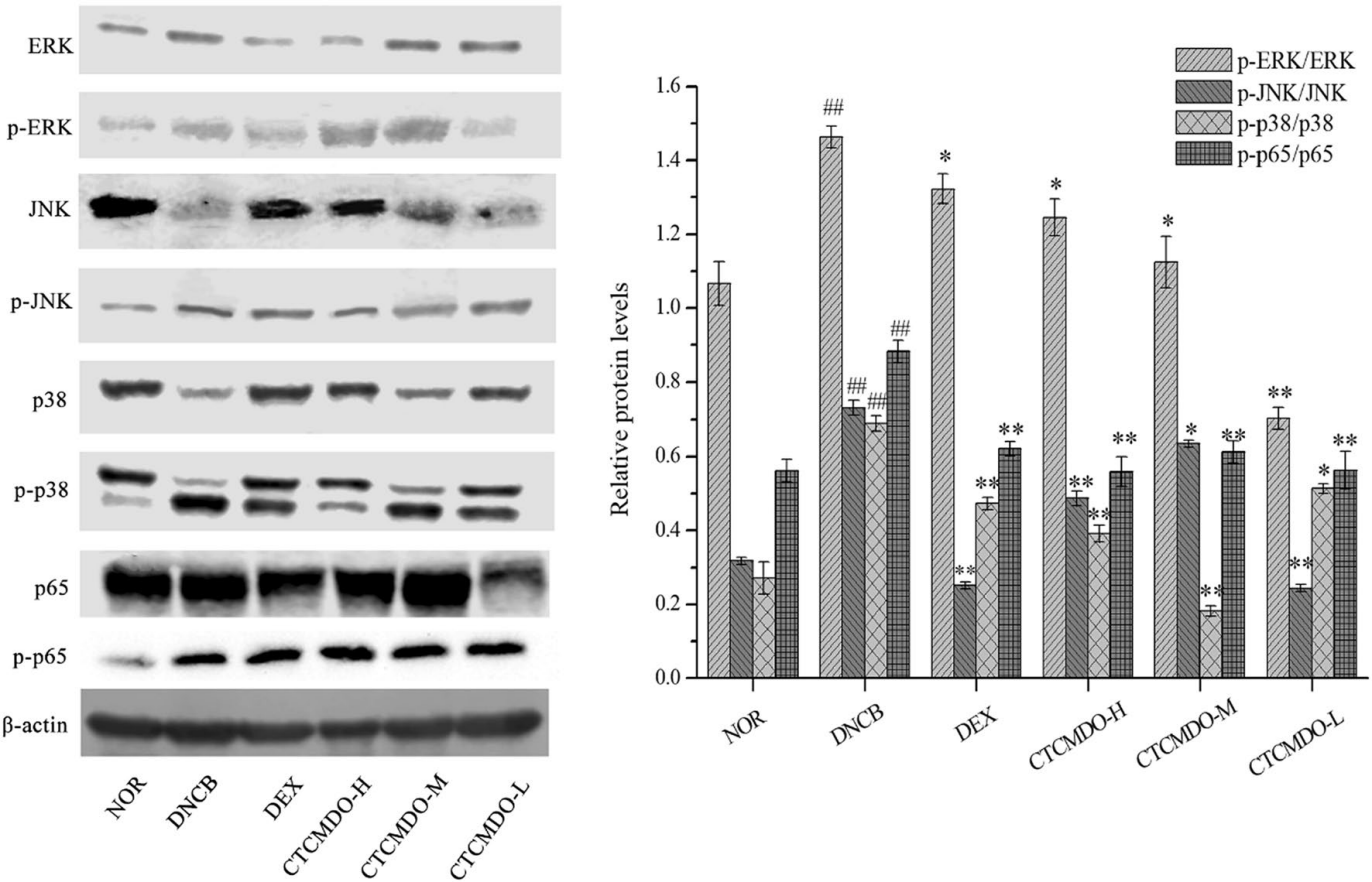
Effects of CTCMDO on DNCB-induced expression levels of proinflammatory markers in mice. ERK, p38, JNK, and NF-κB p65 levels. The proinflammatory expression levels were determined using western blot analysis. The results are representative of three separate experiments. *NOR* normal control, *DNCB* DNCB-sensitized mice, *DEX* dexamethasone (0.1 g/cm^2^) + DNCB-sensitized mice. CTCMDO-L (75 mg/g, crude drug/ointment, 0.1 g/cm^2^) + DNCB-sensitized mice. CTCMDO-M (150 mg/g, crude drug/ointment, 0.1 g/cm^2^) + DNCB-sensitized mice and CTCMDO-H (300 mg/g, crude drug/ointment, 0.1 g/cm^2^) + DNCB-sensitized mice. **P < 0.01, *P < 0.05 compared with the DNCB group, ## P < 0.01 compared with the NOR group

To further verify the anti-inflammatory mechanism, the levels of MAPK (ERK, p38, and JNK) and NF-κB (p65) in the whole tissue lysate were evaluated. As shown in Fig. 11, MAPK was significantly increased when treated with DNCB, while the levels of p-ERK, p-JNK and p-p38 were reduced when treated with DEX. Topical application of CTCMDO could restore p-ERK and p-JNK, accompanied with decrement of p-p38 levels. Compared with the normal group, the expression of NF-κB protein in the skin of the DNCB group was significantly higher (P < 0.01). And the expression of NF-κB p-p65 protein in the skin tissue of the DEX group and the CTCMDO group were lower than that of in the model group.

## Discussion

AD as one kind of complex skin disease is usually induced by complicating factors. Currently, the inflammatory response and immunomodulatory ability of the skin are usually considered to evaluate the therapeutic effect of AD. An AD-like mouse model was usually established by inducing DNCB. The performance of mouse model is considered to be similar to that of clinical patients [31]. In this study, a mixture of 1% DNCB, acetone and olive oil was prepared to establish the model. The experimental results showed that the degree of ear swelling in the model group was significantly increased. And the degree of ear swelling was significantly reduced when treated with CTCMDO and dexamethasone acetate, respectively. Pathological sections results indicated that the back skin of the mice in the model group were severely keratinized, edematous and infiltrated with eosinophils. Granulocyte infiltration was consistent with the results of AD-like models [32], suggesting that the AD-like mice model had been successfully established in this study.

In vivo, the histopathological results showed that thickness of the skin had been obviously improved when treated with CTCMDO. SP consists of 11 kinds of amino acids, which is secreted by specific sensory nerve endings and responds to the various stimuli [33]. A series of pathophysiological processes, such as immune regulation and inflammation, pain and itching can be regulated by the neuropeptide SP. Furthermore, SP also plays a key role in improving skin nerve activity, and had been considered as an important inflammatory mediator to adjust skin neurogenic pruritus. The previous study had shown that SP could mediate some inflammatory reactions, and induce itching, pain and other neural activities through G protein-coupled receptors [34]. Therefore, inhibition of the expression of SP is advantage to relieve the pain, itching and other symptoms during the onset of AD, leading to alleviate the suffering of patients [35]. Therefore, the secretions of SP had been detected. The immunohistochemically experimental results showed that compared with the blank group, the expression of SP in the skin lesions was higher in the AD model group. Compared with the model group, the expression of SP was lower in the skin lesions in the positive control group and the CTCMDO administration groups (P > 0.01), respectively. The above results indicated that CTCMDO can improve itching during the onset of AD via inhibiting the expression of SP.

In AD-like skin, abnormal immune activation of T cells is considered to be related to the overexpression of Th2 cytokines, such as IL-4, IL-5, and IL-13 [36]. IL-4 plays a critical role in the induction of IgE synthesis and promotion of the switch from naive T cells to allergic-type Th2 cells [37]. In this study, the changes of the levels of various inflammatory cytokines in the serum involved in the pathogenesis of AD had been checked. The previous report had proofed that TNF-α and IL-4 are the primary cytokines that inducing an inflammatory response, and the AD severity was associated with their concentrations [38]. INF-γ is a multifunctional cytokine that plays an anti-inflammatory role in the inflammatory response [39, 40]. In the present study, the serum levels of the proinflammatory cytokines TNF-α, IL-4, IL-4, IL-6, IgE and INF-γ were increased in the AD-model mice, which is consistent with the result of a previous study [41]. The experimental results indicated that the serum levels of the proinflammatory cytokines of the AD-model mice were significantly suppressed by treating with CTCMDO. Therefore, the potential mechanism of CTCMDO in treating AD may attributed to the modulation of the inflammatory responses in the infected tissues and cells, which is consistent with the results of the molecular docking analysis.

The MAPK and NF-κB signaling pathways as two main approaches are considered to regulate the release of inflammatory factors in skin lesions. NF-κB is a key transcription factor for regulating Th2 cell differentiation and inflammation [42]. The NF-κB pathway could be activated by the internal/external factors stimulus, leading to a significant increment of the level of inflammatory factors in the skin and serum, and accentuating the inflammatory process [43]. Therefore, inhibition of NF-κB activity is advantage to anti-inflammatory therapy. As shown in Fig. 11, western blot analysis results showed that compared with the DEX group, the NF-κB activity was significantly inhibited when treated with topical application of CTCMDO. Many intracellular inflammatory factors would induce hyper phosphorylation in the MAPK signaling pathway, leading to promote an allergic inflammatory response to further activate NF pathway [44]. Therefore, inhibition of phosphorylation of MAPK pathway can reduce the synthesis of proinflammatory cytokines and intracellular signal transduction, and downregulate the activation of NF-κB, resulting in inhibiting the release of inflammatory factors and reducing the local damage caused by inflammation. These results demonstrated that CTCMDO exhibited significant anti-inflammatory effect via inhibiting MAPK phosphorylation and NF-κB activation. The production of multiple inflammatory factors was effectively blockaded, leading to a series of symptoms caused by inflammatory reactions was alleviated. In addition, the effects of CTCMDO on the regulation of p-p38 were further verified in the MAPK pathway. The results showed that CTCMDO was able to significantly inhibit the expression of p38 mRNA in comparison with the DNCB group, which was consistent with the western blotting results.

Natural medicines or Chinese medicines had shown excellent therapeutic effects due to its multiple targets characters of multiple ingredients [45]. In this study, HPLC–MS and fingerprinting were performed to study the “spectrum–effect” relationship of CTCMDO and to explore the potential therapeutic mechanism. The active ingredients of CTCMDO were analyzed by combined HPLC and LC/MS. A total of 44 compounds were identified, including ferulic acid, berberine, palmatine, catalpol, curcumin, sesamin, sesamol and other chemical components. And the preliminary identification of the effective substances was achieved. The chromatogram of sample 1 (S1) was set as the reference map, and the fingerprint of CTCMDO in 10 batches was established to exhibit 17 peaks. Eight chromatographic peaks were assigned, i.e., berberine, coptisine, palmatine, phellodendrine, ferulic acid, curcumin, sesamol and sesamin. In addition, the molecular docking analysis results showed that among the compounds in the CTCMDO prescription, coptisine, sesamin, berberine, palmatine, curcumin, ferulic acid and sesamol exhibited strong binding ability with the related action pathways, leading to the enhanced immune ability [46]. Furthermore, coptisine, sesamin and palmatine exhibited the most prominent binding ability, which might be the main substances of CTCMDO in the treatment of skin inflammation. The currently studies had shown that the above ingredients exhibited good therapeutic effects in the treatment of AD [47], which is consistent with the advantages of the “multi-component, multiple targets, and multi-pathways” of TCM [48].

## Conclusion

In this study, the efficacy and mechanism of CTCMDO in the treatment of AD has been systematically studied via modern TCM chemistry and molecular biology technology. All the results indicated that CTCMDO can significantly improve AD via inhibiting the activation of two signaling pathways (MAPK and NF-κB), subsequently inhibiting the production of inflammatory factors. Furthermore, CTCMDO exhibited the advantages of multi-ingredients, multi-targets, and multi-pathway treatment in comparison with DEX acetate. Specially, CTCMDO exhibited a unique prevention and treatment effect without obvious adverse reactions or drug tolerance after long-term use. Therefore, this work provides a promising way for the future development of therapeutic agents to prevent and treat AD.

## Data Availability

The data sets supporting the conclusions of this article are included within the article and its additional files.

## Abbreviations

DNCB: 1-Chloro-2, 4-dinitrobenzen
TCM: Traditional Chinese medicine
CAM: Complementary and alternative medicine
HPLC: High-performance liquid chromatography
AD: Atopic dermatitis
IgE: Immunoglobulin E
TNF-α: Tumor necrosis factor-α
INF-γ: Interferon-γ
MAPK: Mitogen-activated protein kinase
NF-κB: Nuclear factor-kappa B
IL-6: Interleukin-6
IL-2: Interleukin-2
IL-4: Interleukin-4
SP: Substance P
